# Immune-Modulatory Effects of Bu-Zhong-Yi-Qi-Tang in Ovalbumin-Induced Murine Model of Allergic Asthma

**DOI:** 10.1371/journal.pone.0127636

**Published:** 2015-06-02

**Authors:** Sien-Hung Yang, Ting-I Kao, Bor-Luen Chiang, Hsing-Yu Chen, Kuang-Hua Chen, Jiun-Liang Chen

**Affiliations:** 1 Department of Traditional Chinese Medicine, Division of Chinese Internal Medicine, Chang Gung Memorial Hospital, Taoyuan, Taiwan; 2 School of Chinese Medicine, Chang Gung University, Taoyuan, Taiwan; 3 Graduate Institute of Biomedical Sciences, Division of Natural Products, Chang Gung University, Taoyuan, Taiwan; 4 Graduate Institute of Immunology, National Taiwan University, Taipei, Taiwan; 5 Department of Pediatrics, National Taiwan University, Taipei, Taiwan; 6 Graduate Institute of Clinical Medical Sciences, College of Medicine, Chang Gung University, Taoyuan, Taiwan; 7 Department of Pathology, Chang Gung Memorial Hospital, Taoyuan, Taiwan; Mie University Graduate School of Medicine, JAPAN

## Abstract

**Background:**

Bu-zhong-yi-qi-tang (BZYQT), an herbal formula of traditional Chinese medicine, has been an effective regimen of allergic diseases for nearly 800 years. Our previous report has demonstrated its anti-inflammatory effects in patients with perennial allergic rhinitis, and the aim of this study is to investigate the anti-asthmatic effect of BZYQT.

**Methods:**

Female BALB/cByJNarl mice were sensitized with normal saline (control group) or OVA. Mice sensitized by OVA were fed with distilled water (OVA group), oral 0.5 g/Kg (low-dose group) or 1 g/Kg (high-dose group) of BZYQT solution once daily on days 36-40 besides their routine diet. Airway hyper-responsiveness (AHR), eosinophil infiltration, levels of cytokines and total immunoglobulin E (IgE) in broncho-alveolar lavage fluid (BALF) were determined. The lungs and tracheas were removed, and histopathologic examination was subsequently performed.

**Results:**

AHR was significantly reduced in both low- and high-dose BZYQT groups compared with the OVA group after inhalation of the highest dose of methacholine (50 mg/ml). The levels of eotaxin, Th2-related cytokines (IL-4, IL-5, IL-13), IgE, and eosinophil infiltration in BALF were significantly decreased in both BZYQT groups compared with the OVA group. Histopathologic examination revealed that eosinophil infiltration of the lung and trachea tissues was remarkably attenuated in both BZYQT groups.

**Conclusions:**

Oral administration of BZYQT solution may exert anti-asthmatic effect by relieving AHR in OVA-sensitized mice, which is compatible with our clinical experience. Although detailed mechanism is to be determined, we surmise that it may be correlated with the immune-modulatory effects of inhibiting Th2 responses on the basis of our limited results.

## Introduction

Asthma has become an important public health problem worldwide, and patients with bronchial asthma are usually characterized with symptoms of wheezing, cough, and shortness of breath [1]. Chronic inflammation of airways has been identified to play a key role in the pathogenesis of bronchial asthma, which used to be taken as a disease of bronchial smooth muscles [2, 3]. Airway inflammation in asthma is characterized by acute onset airway hyper-responsiveness (AHR) and infiltration of inflammatory cells, especially eosinophils [4–6]. Furthermore, eosinophils have been related to the severity of asthma, and several mediators resulting in eosinophil activation may also lead to contraction of airway smooth muscles [7, 8]. The onset of asthma is generally attributed to genetic and environmental factors; however, predominant Th2 cell activity has been identified as part of the core pathogenesis of asthma [9]. Activated Th2 cells will secrete cytokines, such as IL-4, IL-5, and IL-13 [9, 10]. IL-4 has been proved to promote IgE production and T cell differentiation into Th2 cells [11, 12]. IL-5, however, induces the differentiation, maturation, and migration of eosinophils to the local tissue of inflammation [13, 14]. Overproduction of IL-13 enhances airway hyper-responsiveness, and it is also implicated in the pathogenesis of airway eosinophilia [15]. Therefore, suppression of Th2 cytokines may have the potential to alleviate chronic airway inflammation and, subsequently, the symptoms of asthma [10].

Bu-Zhong-Yi-Qi-Tang (BZYQT), composed of ten medical herbs (Table 1), is a well-known formula of traditional Chinese medicine and has been widely used for treatment of allergic diseases [16]. In the past decades, it has been frequently reported that BZYQT possesses a variety of immune-modulatory effects, such as stimulation of peripheral blood mononuclear cells to produce G-CSF and TNF-alpha in Hepatocellular carcinoma (HCC) patients [17], suppression of the proliferation of human hepatoma cell lines by inhibition of DNA synthesis followed by apoptosis [18], suppression of contact hypersensitivity during chronic stage [19], and reduction of IgE levels in the atopic dermatitis animal model [20]. However, none of these previous reports has investigated its effect on relieving clinical airway symptoms, such as AHR, and the potential correlation with Th2 activity and eosinophil infiltration. In view of these facts, this study is designed to determine whether BZYQT alleviates hyper-responsiveness and chronic inflammation of the airways in the asthmatic murine model sensitized by ovalbumin.

**Table 1:** Composition of Bu-Zhong-Yi-Qi-Tang (every 7.56g of water extract are derived from 27g of raw herb).

Plant Name	Plant Part	Ratio
Astragalus mongholicus Bunge.	Root	6.0
Panax ginseng C.A.Mey.	Root	4.0
Glycyrrhiza uralensis Fisch.	Root and rhizome	4.0
Zingiber officinale Rosc.	Rhizome	3.0
Ziziphus jujuba Mill. var. inermis Rehd.	Fruit	2.0
Angelica dah-rica Fisch. ex Hoffm.	Root	2.0
Citrus reticulata Blanco.	Pericarp	2.0
Atractylodes macrocephala Koidz.	Rhizome	2.0
Bupleurum chinense DC.	Root	1.0
Cimicifuga foetida L.	Rhizome	1.0
Total		27.0

## Materials and Methods

### Animals and Ethics Statement

Inbred female BALB/cByJNarl mice aged 6–8 weeks and weighed around 15–20 g were purchased from the National Laboratory Animal Center (Taipei, Taiwan) [21]. All mice were housed and maintained at the animal center of Chang Gung University before the experiment. The experimental protocols used in this study were approved by the Institutional Animal Care and Use Committee of Chang Gung Memorial Hospital (Approval ID: AN-94082).

### Preparation of bu-zhong-yi-qi-tang (BZYQT)

**Fig 1:**
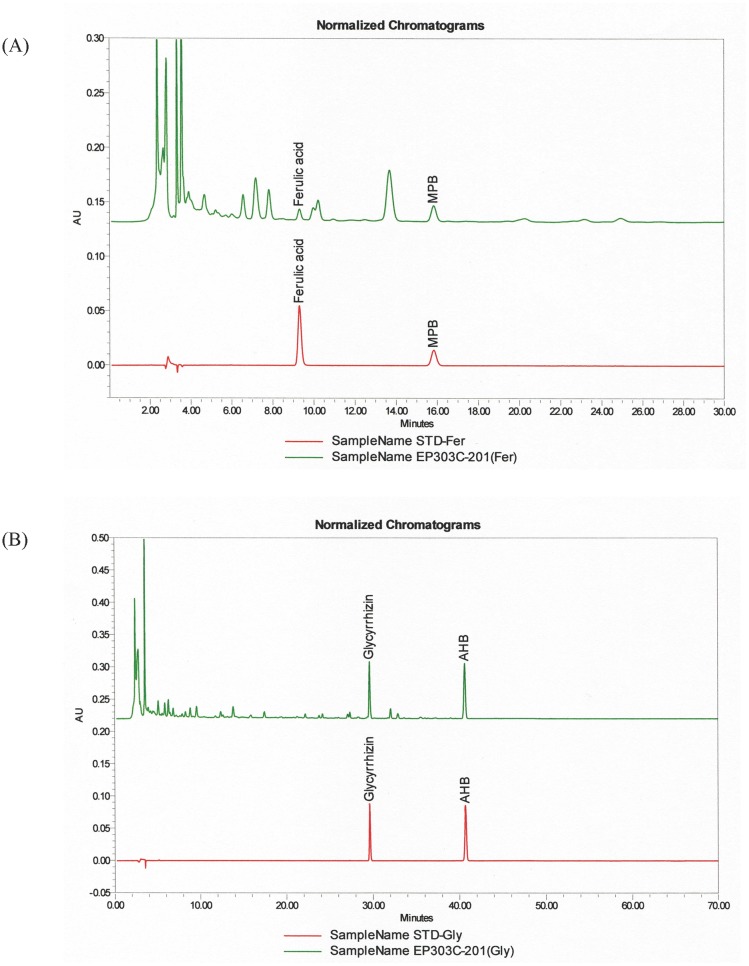
HPLC data of Bu-Zhong-Yi-Qi-Tang (BZYQT) (Upper: BZYQT; Lower: standard sample). The peaks indicate the existence of glycyrrhizin, AHB, Ferulic acid, and MPB in both panels, which proves the authenticity of BZYQT.

BZYQT was purchased from the GMP pharmaceutical factory of Chuang Song Zong Pharmaceutical Co. (Kaohsiung, Taiwan), and the authenticity was proved by HPLC (Fig 1). The original preparation is concentrated powder of the aqueous-extract mixture of 10 common Chinese herbal plants, which are listed in Table 1[16]. The concentrated powder of BZYQT was dissolved (1 g/ml) in distilled water at 26°C, and was ready for administration to mice via gastric tube.

### Mice sensitization, challenge, and drug administration

A total of 30 female BALB/c(ByJNarl) mice were randomly divided into 4 groups:

1. Control group (n = 10): sensitized and challenged with normal saline, and treated with distilled water
2. OVA group (n = 4): sensitized and challenged with OVA, and treated with distilled water
3. Low-dose BZYQT (LD) group (n = 8): sensitized and challenged with OVA, and treated with 0.5 g/Kg of BZYQT solution
4. High-dose BZYQT (HD) group (n = 8): sensitized and challenged with OVA, and treated with 1 g/Kg of BZYQT solution

Mice in OVA and BZYQT groups were sensitized by three intra-peritoneal (I.P.) injections of 25 μg OVA (grade V; Sigma, St. Louis, USA), which had been emulsified in 4 mg aluminum hydroxide in 100 μl normal saline on days 0, 14, 28 [22]. Mice in the control group were injected with 100 μl normal saline. Mice were challenged with 100 μg OVA dissolved in 50μl normal saline (OVA group and BZYQT groups), or with 50 μl normal saline alone (control group), intra-nasally (I.N.) on days 42–44. Mice in each group were fed once daily with distilled water (control group and OVA group), or with solution of BZYQT (low-/ high-dose BZYQT groups) via gastric tube on days 36–40 besides their routine diet.

### Measurement of airway hyper-responsiveness (AHR)

AHR was measured on day 45, 24 hours after last OVA challenge, with stimulation of methacholine to conscious, unrestrained mice in plethysmograph (Buxco Electronics, Troy, NY, USA). Mice in each group were exposed to a series of incremental dosages of aerosolized methacholine (6.25, 12.5, 25, 50 mg/ml) for one minute, and AHR was recorded as enhanced pause (Penh), which was calculated with the program provided by Buxco Electronics [22]. Enhanced pause (Penh) represents airway hyper-responsiveness and is defined as pause × (PEP/PIP) (pause, time of expiration; PEP, peak expiratory pressure; and PIP, peak inspiratory pressure) [23].

### Collection of broncho-alveolar lavage fluid (BALF)

Mice were sacrificed with the cervical dislocation method on the day following AHR measurement (Day 46). To collect BALF, lungs of each mouse were lavaged with 1 ml of normal saline for three times. The different cell types and numbers were determined after cytospin preparations and Liu stain of BALF. The percentage of eosinophil was determined on the basis of eosinophil cell counts in 500 BALF cells according to morphology and staining characteristics. After centrifugation, the supernatant of BALF was collected, frozen and stored at -80°C until further cytokine analysis [22].

### Determination of cytokines, IgE, and chemokine levels in BALF samples

BALF samples were diluted to either 1:2(for cytokine analysis), or 1:4 (for eotaxin analysis). The concentration of IL-4, IL-5, IL-13, and eotaxin in BALF samples were measured with enzyme-linked immunosorbent assay (ELISA) kits (R&D Systems, Minneapolis, MN, USA) according to the manufacturer’s protocols. Total immunoglobulin E (IgE) levels in BALF samples were also measured by ELISA, directly using OD 450 nm readings.[22, 24]

### Histopathologic examination of lungs

After BALF collection, the lungs and tracheas were removed from mice, and were fixed with 10% of buffered formalin for one week. After they were fixed, the specimens were embedded in OCT (Tissue-Tek, Sakura Finetek, Torrance, CA, USA)[24]. Each tissue specimen was sliced into 6-μm sections, and was stained with H&E method for light microscopic inspection.

### Statistical analysis

The results are presented in mean ± SD (standard deviation). The statistical significance was determined by one-way analysis of variance (ANOVA) with a Bonferroni post-hoc test, and a difference with P-value < 0.05 was considered significant.

## Results

### Oral administration of BZYQT attenuated OVA-induced AHR

**Fig 2:**
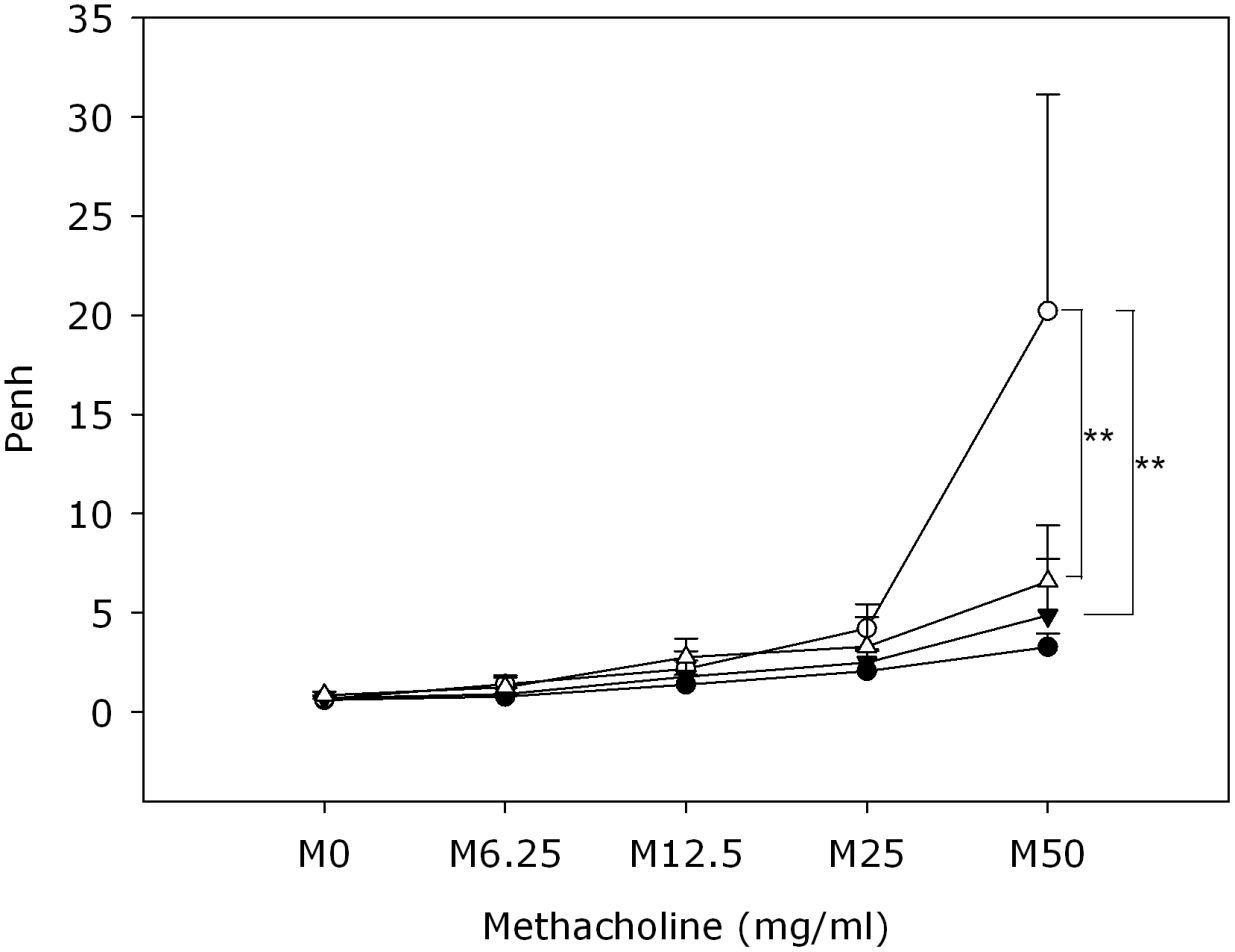
Effect of oral administration of BZYQT on airway hyperresponsiveness (AHR). After last OVA challenge, mice were exposed to a series of incremental dosages of methacholine (6.25, 12.5, 25, 50 mg/ml). The Penh values were then recorded, indicating AHR of mice under each dosage of methacholine stimulation. Control group (●, n = 10), OVA group (○, n = 4), Low dose (0.5 g/Kg) BZYQT group (△, n = 8), High dose (1 g/Kg) BZYQT group (▼, n = 8). Data were presented in mean ± standard deviation. ** p< 0.01 compared with the OVA group.

The mice in low-/high-dose BZYQT groups were administered with solution of BZYQT once daily on days 36–40 in order to determine the effect of BZYQT on OVA-induced airway inflammation. AHR was determined in all mice after inhalation of a series of dosages of methacholine ranging from 6.25 to 50 mg/ml. There were no significant differences in AHR observed between the OVA group and the BZYQT groups with the inhalation of 0-25mg/ml methacholine. The highest dose of methacholine (50 mg/ml) inhalation, however, induced significantly higher AHR in the OVA group than that in the control group, low-dose BZYQT group, and high-dose BZYQT group (Fig 2). Moreover, there was no significant difference between Penh values of both low- and high-dose BZYQT groups after the highest level (50mg/ml) of methacholine stimulation. These findings indicated that both low- and high-dose BZYQT treatment might significantly suppress OVA-induced AHR with similar potency.

### Effect of BZYQT on Th2-related cytokines, eotaxin and IgE levels in BALF

**Fig 3:**
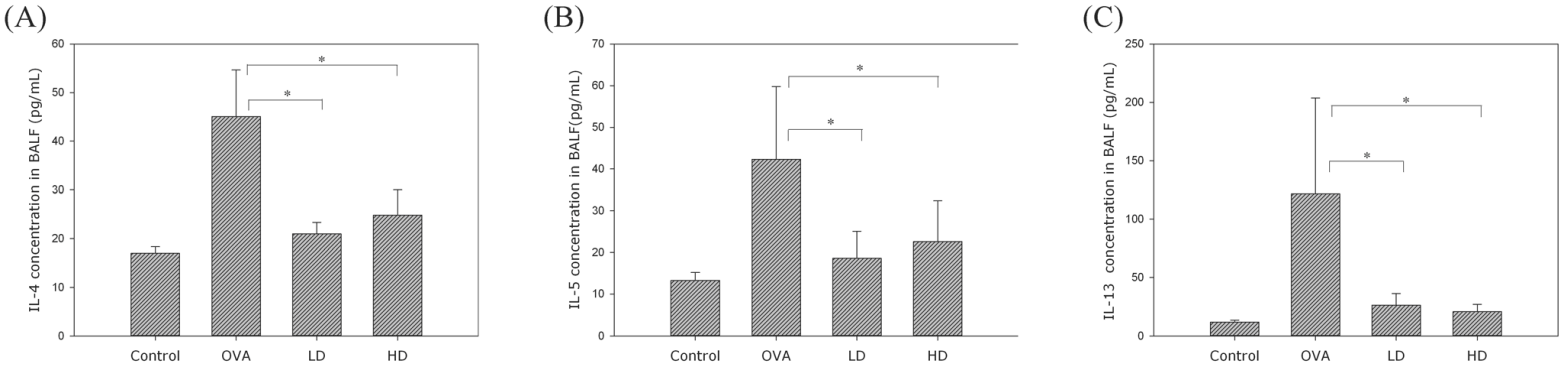
BZYQT regulated Th2 cytokines in BALF of asthmatic murine model sensitized by OVA. The concentrations of IL-4 (A), IL-5 (B), and IL-13 (C) were determined by ELISA. Data were presented in mean ± standard deviation. * p < 0.05 compared with the OVA group.

**Fig 4:**
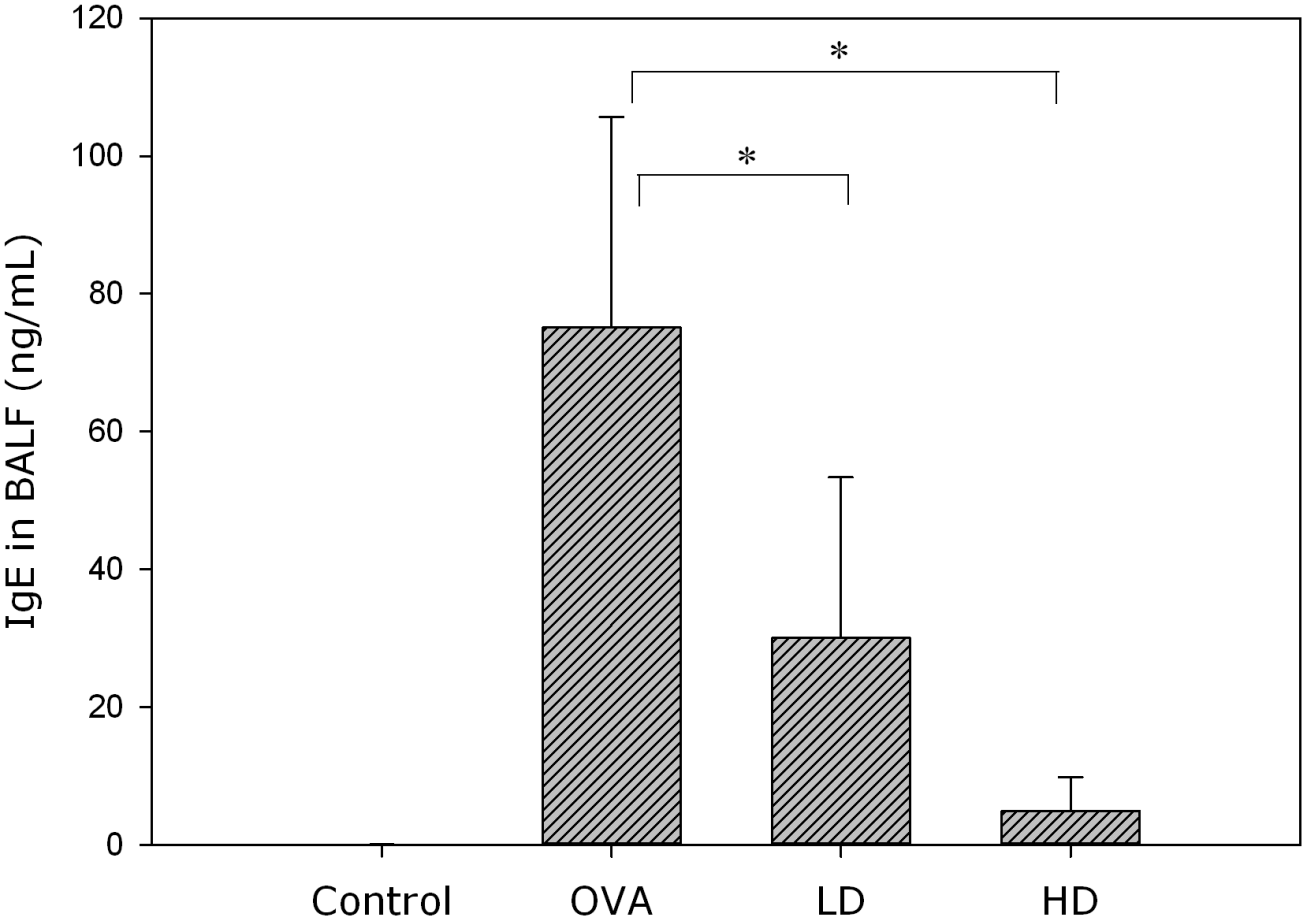
BZYQT significantly attenuated IgE levels in BALF of OVA-sensitized mice. These data were measured by ELISA, and presented in mean ± standard deviation. ** p < 0.01 compared with the OVA group.

**Fig 5:**
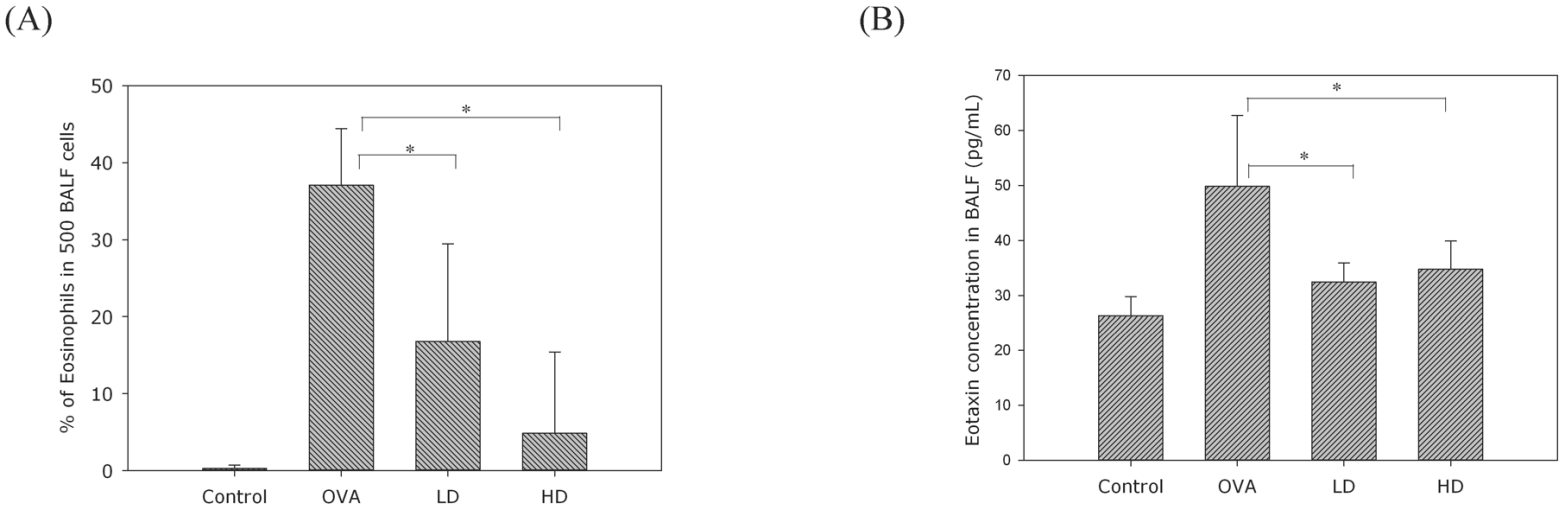
The concentration of eosinophil chemoattractant eotaxin (B) and the percentage of eosinophil cell count (A) in BALF were significantly down-regulated in the BZYQT groups. Data were presented in mean ± standard deviation. * p < 0.05 compared with the OVA group.

All the mice were sacrificed on day 46, and BALF was collected from their lungs. After centrifugation, the supernatant of BALF was analyzed for levels of Th2-related cytokines (IL-4, IL-5, IL-13), eotaxin and IgE with ELISA. The two BZYQT groups were found to have significantly lower levels of IL-4, IL-5, IL-13 (Fig 3), IgE (Fig 4), and eotaxin (Fig 5B) than the OVA group.

### BZYQT attenuated OVA-induced eosinophil infiltration in BALF

To evaluate the effect of BZYQT on eosinophil infiltration, BALF samples after cytospin preparations were stained with Liu stain, and the percentage of eosinophil was determined on the basis of eosinophil cell counts in 500 BALF cells according to morphology and staining characteristics. The percentage of eosinophil was significantly higher in the OVA group than the control group, and it was significantly attenuated in both BZYQT groups (Fig 5A).

### Histopathologic examination of effect of BZYQT on eosinophil infiltration

The lungs and tracheas were removed, fixed with formalin, sliced into sections, and stained with H&E method. For each animal, the extent of the lung infiltrate was determined by establishing the percentage of compromised bronchioli within 30 of such structures, randomly selected at low-power fields (with a X4 objective). In addition, with the X40 objective, 8random digital images per group were taken within areas of overt peribronchiolar inflammation. Total eosinophil cell counts were determined from these images and expressed as number of cells per square millimeter. Such quantification was focused on peribronchiolar areas that were the main sites of inflammatory reaction.

**Fig 6:**
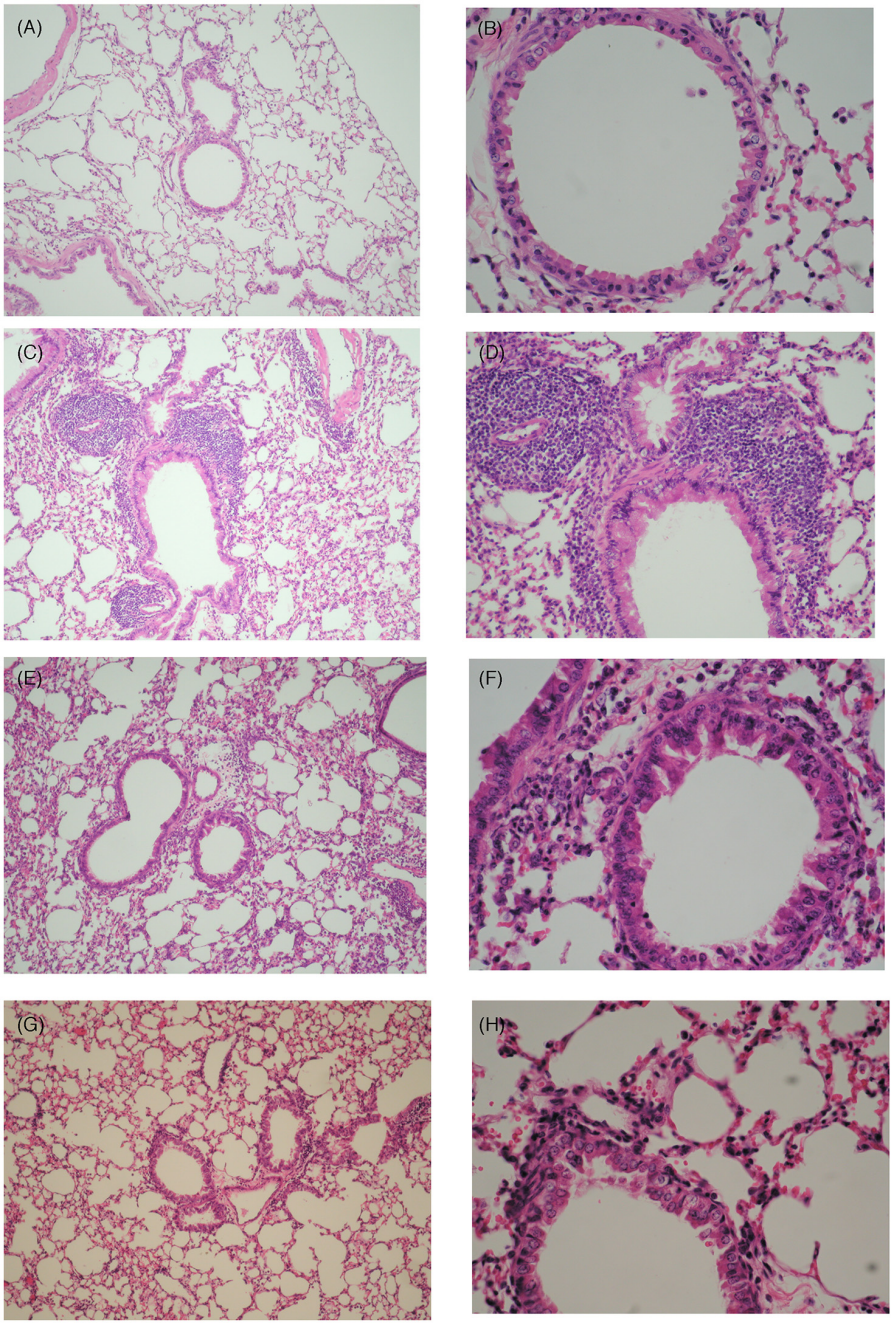
Histopathologic examination of sections of lung and trachea tissues with H&E stain. Specimens were derived from the control (low power view, A; high power view, B), OVA (low power view, C; high power view, D), LD (low power view, E; high power view, F), and HD (low power view, G; high power view, H) groups, respectively. Notably, eosinophil infiltration over the perivascular and peribronchiolar areas were enhanced in OVA group, and then attenuated in both BZYQT groups significantly.

Histopathologic examination of the specimens revealed severe infiltration of eosinophils over the perivascular and peribronchiolar areas of the lung (45,2±5.4/HPF) and trachea tissues of the OVA group (Fig 6C and 6D). Oral administration of low-(23.8±4.2/HPF) and high-dose (5.1±3.9/HPF) BZYQT solutions, however, significantly reduced eosinophil infiltration into lung tissues (Fig 6).

## Discussion

The pathophysiology of asthma is extremely complicated, involving the network of Th2 cells and cytokines, and airway inflammation has been proved to be a major feature of the pathogenesis of chronic asthma. After exposure to allergens, Th2 cells secrete cytokines, such as IL-4, IL-5, and IL-13 [25], which promote airway hyper-responsiveness, IgE production, and infiltration of inflammatory cells, such as eosinophil, into the airways [26]. These pathophysiological changes were confirmed in the OVA-sensitized mice in our study. On the other hand, research on cytokine-related gene knockout mice has demonstrated that the symptoms of asthma may be aggravated by Th2 cytokines [27, 28].

Our study evaluated the beneficial effects of BZYQT on airway hyper-responsiveness and Th2 overexpression in OVA-sensitized murine model of asthma. The levels of inflammatory cell (eosinophil) infiltration, Th2 cytokines (IL-4, IL-5, IL-13), eosinophil-related chemokine (eotaxin), IgE and AHR were all significantly enhanced in the OVA-sensitized group. However, all the above levels were remarkably reduced in both low- and high-dose BZYQT groups.

IL-5 plays a key role in the terminal differentiation, maturation, and survival of eosinophils, and together with the chemokine eotaxin, promotes eosinophil recruitment to the site of inflammation [12, 14]. It has been reported that murine model which is in lack of IL-5 expression would not develop AHR or eosinophilia though under allergen challenge[29, 30]. Anti-IL-5 regimens may ameliorate airway allergic inflammation and reduce eosinophils in the blood and sputum; however, AHR and late allergic responses were not significantly improved[29–31]. Recent studies has revealed that anti-IL-5 regimens may reduce frequency of exacerbation in severe asthma more significantly in subgroups of patients, in which eosinophils play a pathogenic role[32–34]. In our study, BZYQT has been demonstrated to decrease the levels of IL-5 and the number of eosinophil in BALF and in the lung tissues surrounding the airways as well. Although further studies are needed to clarify the detailed mechanism, we suspect that BZYQT exerted anti-asthmatic effect through down-regulating Th2 response, especially IL-5 expression, and eosinophilic inflammation was ameliorated subsequently.

IL-4 facilitates the differentiation and proliferation of Th2 cells, the switching of B cells to secrete IgE, and the development of AHR as well [12, 34]. On the other hand, inhibition of IL-4 by monoclonal antibodies attenuates airway eosinophilia and IgE level in allergic mice [35]; therefore, the production of IgE is closely related to the concentration of IL-4. Furthermore, it has been reported that IL-4 deficient mice demonstrated to be spared from asthmatic attack and airway inflammation[36]. Th2 cytokines, especially IL-4 and IL-13, are crucial mediators of asthma, and both are proved to play an important role in up-regulating the eosinophil chemo-attractants of eotaxin [37]. There is evidence showing that eosinophilia may be correlated with the induction of AHR and the severity of asthma [8, 38]. BZYQT has been used in traditional Chinese medicine for allergic asthma treatment and prevention. Besides relieving AHR, which we have already known clinically, we demonstrated its efficacy in reducing IL-4 and IgE levels in BALF in murine model of allergic asthma. Moreover, histopathologic analysis also confirmed that administration of BZYQT may significantly reduce eosinophil infiltration into the perivascular and peribronchiolar areas of the lung and trachea tissues. Though further evidence may be need, it is indicated in our study that BZYQT reduced the levels of IgE of OVA-sensitized mice by down-regulating IL-4, and its suppression of IL-4 and IL-13 may also contribute to relieve lung eosinophila and AHR.

Bu-Zhong-Yi-Qi-Tang (BZYQT), which comprises crude ingredients extracted from ten herbs, has been a well-known traditional Chinese medicine formula used in preventive therapy for allergic diseases during non-acute stages for 800 years. BZYQT has been proved to have immune-modulatory effects on allergic diseases. A previous clinical study had revealed that long-term therapy with BZYQT suppresses total serum levels of IgE, PGE2, LTC4, and COX-2 mRNA expression in IL-4-stimulated PMN in patients with perennial allergic rhinitis, indicating that BZYQT is beneficial to the non-acute stage of allergic rhinitis via anti-inflammatory mechanisms [16]. Moreover, BZYQT can also decrease the IgE level in serum of mice by inhibiting the development of IL-4-producing CD4+ T cells [39]. Another study had revealed that oral administration of BZYQT suppresses IgE antibody production and histamine release in type I allergic reaction in mice immunized with ovalbumin, revealing the potential efficacy of BZYQT in treating type I allergic diseases, such as asthma [40]. Ishimitsu had been reported a similar study revealed that BZYQT suppresses IgE and Th2-type cytokine production [41]. Consistent with previous findings, our results demonstrated that BZYQT alleviated airway eosinophilia and hyper-responsiveness, which may be accomplished by decreasing the concentration of Th2 cytokines, eotaxin, and IgE in OVA-sensitized mice. However, due to the complexity of the component of Chinese medical formula, it remains unclear whether a specific compound inside acts alone or multiple compounds may interact to achieve this efficacy we demonstrated in this study. It is anticipated that future studies may reveal the molecular mechanism of the immune-modulatory effects of BZYQT.

## Conclusions

Oral administration of BZYQT demonstrated immune-modulatory effects by ameliorating AHR and airway eosinophilia in the murine model of allergic asthma induced by ovalbumin, and those effects may be accomplished by down-regulating Th2 responses. However, further studies are needed to clarify its underlying mechanism.
